# Stress barriers controlling lateral migration of magma revealed by seismic tomography

**DOI:** 10.1038/srep40757

**Published:** 2017-01-13

**Authors:** J. Martí, A. Villaseñor, A. Geyer, C. López, A. Tryggvason

**Affiliations:** 1 Institute of Earth Sciences Jaume Almera, ICTJA-CSIC, Barcelona, Spain; 2 Observatorio Geofísico Central, Instituto Geográfico Nacional (IGN), Madrid, Spain; 3 Department of Earth Sciences, Geophysics, Uppsala University, Uppsala, Sweden

## Abstract

Understanding how monogenetic volcanic systems work requires full comprehension of the local and regional stresses that govern magma migration inside them and why/how they seem to change from one eruption to another. During the 2011–2012 El Hierro eruption (Canary Islands) the characteristics of unrest, including a continuous change in the location of seismicity, made the location of the future vent unpredictable, so short term hazard assessment was highly imprecise. A 3D P-wave velocity model is obtained using arrival times of the earthquakes occurred during that pre-eruptive unrest and several latter post-eruptive seismic crises not related to further eruptions. This model reveals the rheological and structural complexity of the interior of El Hierro volcanic island. It shows a number of stress barriers corresponding to regional tectonic structures and blocked pathways from previous eruptions, which controlled ascent and lateral migration of magma and, together with the existence of N-S regional compression, reduced its options to find a suitable path to reach the surface and erupt.

Observation of unrest episodes preceding recent volcanic eruptions^1^,^2^,^3^,^4^ offer increasing evidence of effective lateral migration of magma at different depths inside the volcanic system, thus confirming earlier studies that suggested such magma behaviour^5^,^6^,^7^. Petrological evidence also supports that subhorizontal transport of magma within the lithosphere is a common process that contributes to the growth of the volcanic edifice and to the location of new eruption vents^8^,^9^. Moreover, dyke intrusions not ending with an eruption have been tracked in different rift systems^10^,^11^,^12^.

Magma movement and accumulation is controlled by local stress barriers that mainly correspond to rheological and/or structural discontinuities present in the volcanic edifice^13^,^14^,^15^. Rheological discontinuities may be defined by stratigraphic contacts between materials of different lithologies (i.e., different stiffness) or by intrusive contacts between solidified magma and the host rock, while structural discontinuities may correspond to fault planes or tensile fractures. Magma always tries to spend the minimum energy when it migrates inside the volcanic edifice. This means that it will tend to follow a path normal to the minimum compressive stress, i.e. parallel to the maximum compressive stress. If in its ascent to the surface magma finds a rheological or structural contrast between rocks, magma may become arrested or intrude laterally forming a sill^15^,^16^.

In monogenetic volcanism each eruption occurs from a different vent and generates a new volcanic cone^17^,^18^. What causes magma to follow a different path in each eruption is still unclear, although this is of fundamental importance to conduct hazard assessment in monogenetic volcanic fields. It is obvious that some kind of tectonic control must exist, as demonstrated by the tendency of magma to erupt along main tectonic lineaments^17^. However, the key parameters determining the exact location for future eruptions are not evident.

The eruption of El Hierro (Canary Islands) in 2011–2012 provided good evidence for lateral migration of magma during three months before the onset of the eruption^2^,^19^ (Fig. 1). In this particular case, magma migrated more than 20 km from north to south below the island at a depth of 12–15 km, corresponding with the mantle-crust discontinuity. In comparison with other examples occurred in rift zones (e.g.: Iceland, Afar), where magma migrated more or less linearly following the main structural trends of the rifts structures^4^,^10^,^11^,^12^, the lateral migration of magma at El Hierro was clearly affected by pre-existing structures that defined stress barriers that controlled its journey inside the volcanic edifice^2^,^19^. A similar pattern of lateral magma migration has been observed in some of the six post-eruptive unrest episodes that have occurred since 2012 (Fig. 2).

A detailed 3D P-wave velocity model obtained using local-earthquake arrival times from all seismicity occurred in El Hierro since April 2011, which includes all unrest episodes, reveals clues to understand why magma did not follow a straight path, but described a rather irregular trajectory avoiding areas occupied at surface by previous volcanic edifices. Our results explain the particular case of El Hierro but show, in a more general way, how stress barriers created by structural or rheological contrasts control magma migration, thus explaining one of the main features of monogenetic volcanism.

## Geologic setting and seismicity during volcanic unrests at El Hierro

**Figure 1:**
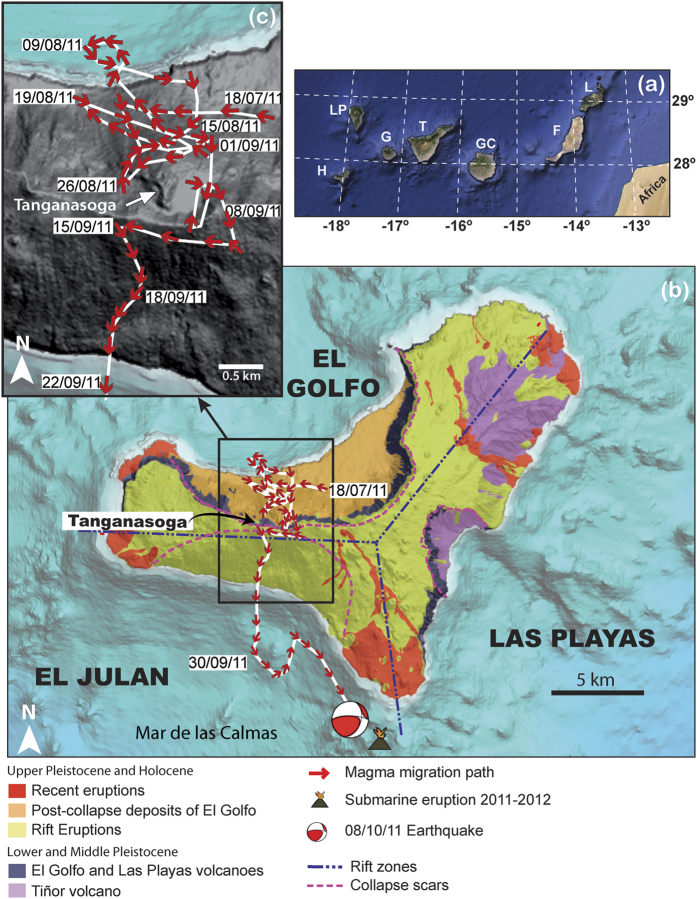
(**a**) Location map of the Canary Islands. (**b**) DEM of El Hierro island showing the evolution of daily average epicentral location of seismic events with estimated location errors smaller than 3 km. Dates are in dd/mm/yy. Location and focal mechanism of the earthquake preceding the onset of the eruption and location of the vent are also shown. Black dashed lines: trace of the rift zones. Red dashed lines: trace of landslides scars. (**c**) Zoom of the area highlighted. See Fig. 3 for location of seismic stations. (Modified after Martí *et al*.^2^ and created using Adobe Illustrator CS5.1 (Copyright © 1984–2016 Adobe Systems Incorporated and its licensors); Reproduced with permission of John Wiley & Sons).

El Hierro is the youngest of the Canary Islands, a group of seven volcanic islands located a few hundred kilometres off the northwest coast of Africa (Fig. 1). El Hierro Island is situated at the south-western corner of the archipelago with the oldest subaerial rocks dated at 1.12 Ma^20^. El Hierro rises from 4,000 m depth below sea level to an altitude of about 1,500 m above sea level and has an estimated volume of about 5,500 km^3^. It is formed by two volcanic edifices that developed successively: the Tiñor volcano in the NE and El Golfo-Las Playas volcano in the NW (see Fig. 1). El Hierro includes three rift zones that are separated by the collapse scars of El Golfo, Las Playas, and El Julan to the NW, SE, and SW, respectively^20^,^21^ (Fig. 1). The emerged parts of these rifts are defined by narrow and steep topographic ridges corresponding to aligned dyke complexes with clusters of cinder cones. Pre-historical eruptions have been recognised on all three rifts of El Hierro.

From petrological studies, it is inferred that mafic eruptions in the Canaries are fed by single batches of asthenospheric magmas that ascend towards the surface from different storage regions located at various depths below the islands^22^,^23^. It has been suggested that the plumbing system beneath El Hierro is characterised by a multi-stage ascent of magmas with major fractionation occurring within the uppermost mantle^24^. In this model, incoming magma batches would also become mingled or thoroughly mixed with other ascending batches. The base of the crust below at El Hierro is assumed to be at a depth of 12–15 km^25^,^26^.

Intense seismicity, surface deformation, and gas emissions started in El Hierro on 19 July 2011^19^. Before the onset of the eruption on 10 October 2011 the Spanish Geographic Institute (IGN) (www.ign.es), responsible for volcano monitoring in Spain, recorded nearly 11,000 seismic events with magnitudes up to 4.3 (Fig. 1). Most events were located below El Hierro volcanic edifice, around 12–15 km depth. GPS local network stations showed an accumulated surface horizontal deformation as much as 40 mm. High rates of CO_2_ flux were also measured in the area where epicentres concentrated on-land. During the first two months of unrest, it was observed that surface deformation always preceded a new cluster of seismic events and that periods of high seismic activity alternated with relatively calm periods. From 20 September, the process accelerated abruptly, increasing the total seismic energy released and surface deformation. A seismic event with magnitude 4.3 occurred 30 hours before the onset of the eruption at a depth of 14 km. The eruption began on 10 October at 4:30 am along a fissure, oriented N20E and located on the southern flank of the island, at a depth of 900 m below sea level, and ended at the beginning of March 2012^2^ (Fig. 1).

The distribution of the hypocentres and the characteristics of the seismic events allowed determining the position of magma at each moment during the unrest episode and its evolution until reaching the eruption site (Fig. 1)^2^,^27^. The hypocentres showed how the horizontal position of the seismic events changed continuously, with slight variations in depth^2^,^27^. During the first 50 days of unrest, seismicity concentrated in the north, in the El Golfo area, between the coast and the northern flank of the Tanganasoga volcanic edifice (Fig. 1). Migration of hypocentres during this period defined a very erratic path, as if the magma was not able to find a place to accumulate or a path to migrate. After that period, and before finding a suitable path to reach the surface, magma travelled below El Hierro for more than 14 km from north to south, describing a straight path in some sectors but also turning around some zones such as the Tanganasoga volcano or also around what appears to be another volcanic edifice from the bathymetric data at the south (Fig. 1).

**Figure 2:**
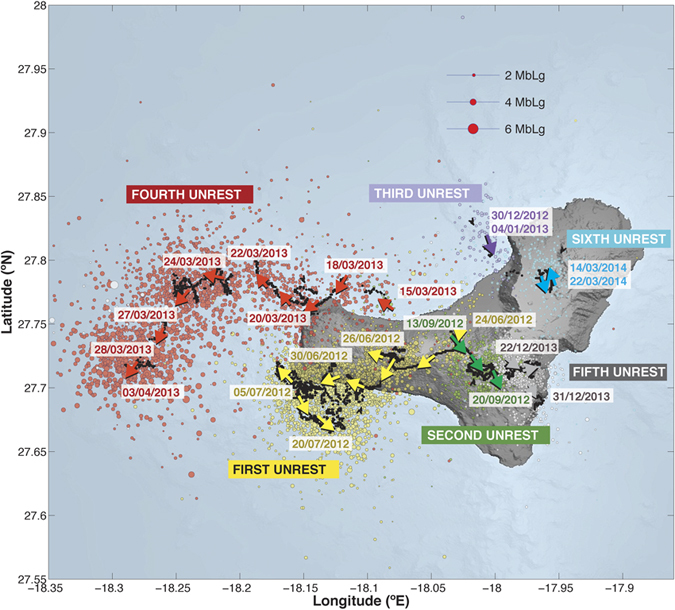
DEM of El Hierro island showing the evolution of daily average epicentral location of seismic events with estimated location errors smaller than 3 km during the post-eruptive unrest episodes. Dates are in dd/mm/yyyy. Figure was created using GMT 5.3.1 software^55^ (http://gmt.soest.hawaii.edu/projects/gmt).

After the eruption, six new unrest episodes, none of them ending with an eruption, occurred: 1st: from 26 June to 20 July 2012; 2nd: from 13 September to 20 September 2012; 3rd: from 30 December 2012 to 4 January 2013; 4th: from 15 March to 3 April 2013; 5th: from 22 December to 31 December 2013; and 6th: from 14 March to 22 March 2014 (IGN web page, www.ign.es/ign/resources/volcanologia/HIERRO.html) (Fig. 2). All these reactivation episodes were characterized by high seismicity and surface deformation affecting different parts of the island, showing in some cases changes in the location of seismicity similar to those observed during the pre-eruptive unrest (Fig. 2).

## Data

**Figure 3:**
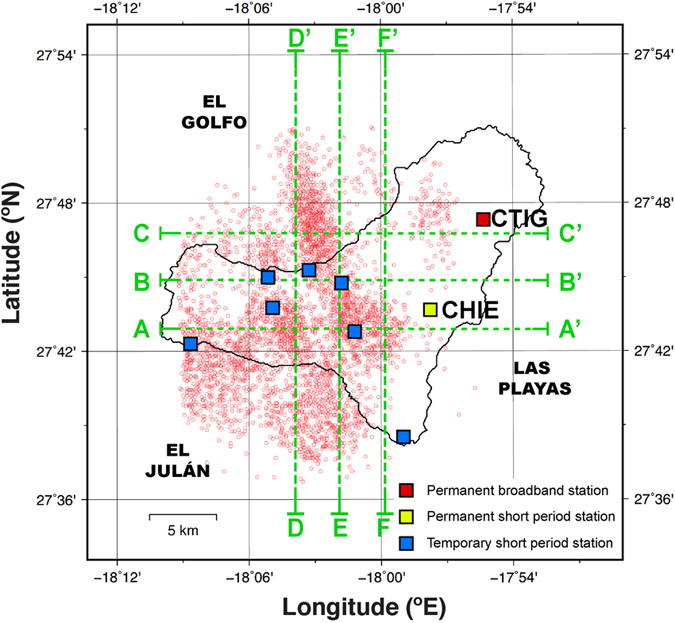
Map view of the 40 × 40 km^2^ model region centred in El Hierro island showing the location and type of seismic stations (colour squares), and selected earthquakes (circles with red outline) used in the tomography. Figure was created using GMT 5.3.1 software^55^ (http://gmt.soest.hawaii.edu/projects/gmt).

The data used in this study consists of arrival times of first-arriving P and S phases recorded at seismic stations in El Hierro. At the time of this writing the El Hierro network consists of a total of 9 stations: 1 permanent broadband station (CTIG), 1 permanent short period station (CHIE) and 7 temporary short period stations deployed in 2011 to monitor the seismic crisis (Fig. 3). Although the seismicity associated with the unrest began on 19 July 2011, we have only considered events from 15 September 2011 (when most of the stations of the temporary network were deployed) to 31 March 2014. This time period (~2.5 years) includes part of the unrest preceding the eruption in 2011–2012, the eruption, and 6 major post-eruptive unrest episodes that were not associated with eruptions (Fig. 2). These later unrest episodes migrated in location, resulting in a rather complete coverage of the island, except for the north-eastern part (Fig. 2).

For the local earthquake tomography we selected well recorded earthquakes inside a model area of 40 × 40 km^2^ centred in the island (Fig. 3), with a maximum focal depth of 35 km. The selection criteria used were: each earthquake must have at least 7 P phases and 7 S phases, P residuals smaller than 0.25 s, S residuals smaller than 0.50 s, and azimuthal gap smaller that 270°. We used gap values greater than the commonly used value of 180° following Koulakov^28^ who has shown that events outside the network can improve the images obtained from local earthquake data. Because of the large number of earthquakes of the series (>20,000 in the IGN catalogue until 2016-01 inclusive; IGN, 2016) and to avoid oversampling the most active regions, we have further added the constraint that only the best-recorded earthquake inside each 0.5 × 0.5 × 0.5 km^3^ cell is selected. This resulted in a total of 3,807 earthquakes with ~31,500P arrival times (Fig. 3).

## Method

The tomographic inversion method used in this study is based on Benz *et al*.^29^ as modified by Tryggvason *et al*.^30^, in order to include P- and S-wave arrival times. It has been successfully applied to different spatial scales, including volcanoes^29^,^31^,^32^ and to larger regions^33^. In this technique, arrival times of first-arriving P and S waves are inverted to simultaneously determine the three-dimensional P- and S-wave velocity structure and earthquake relocations. This non-linear problem is linearized and solved iteratively. In each iteration, updated velocity model and earthquake locations are obtained and used as starting values for the next iteration. The iteration procedure is stopped after a pre-set number of iterations (in our case 10 iterations) or when the data misfit is not reduced significantly. Theoretical travel times are calculated using the finite-difference code of Podvin and Lecomte^34^, which produces accurate travel times even in media with large lateral velocity perturbations and extreme topography, such as volcanic systems. Velocity anomalies with respect to a reference model are solved using the iterative LSQR method of Paige and Saunders^35^ with smoothing constraints applied to control the degree of model roughness. We have tested a wide range of values of the smoothing parameter *k* (see Equation 7 in Benz *et al*.^29^ for its definition) in order to find the optimum value that produces both a relatively smooth model together with a significant reduction in data misfit. We have tested values of *k* ranging from 200 (overdamped) to 10 (underdamped). Results show that decreasing the smoothing parameter down to a value of 50 improves the data fit. Smaller values result in very rough models and in oscillations of the data fit with increasing iterations. Therefore, we have adopted a value of *k* = 50, because it is the one that produces the best variance reduction without degrading the appearance of the model. A more detailed description of these tests is shown in the Supplementary material, together with examples of under- and over-damped models.

The reference/starting model considered here is the 1-D layered model used by the IGN for routine earthquake location in the Canary Islands (see Dañobeitia *et al*.^36^). The target region (model volume) has horizontal dimensions of 40 × 40 km^2^ and it extends from 2 km above sea level to 40.2 km depth, in order to include all possible ray paths of the selected dataset. The velocity model is parameterized as a regular grid of constant velocity cells of 0.8 × 0.8 × 0.8 km^3^ (in the x, y and z directions), resulting in a total of 132,500 model parameters. A finer grid of 0.1 × 0.1 × 0.1 km^3^ was used for the finite-difference travel time calculations. The values of the model cell size and smoothing constraint were determined through a series of empirical tests^29^. Our goal was to determine the smallest cell size that could be well resolved by the ray-path coverage, and the lowest smoothing constraint that would result in stable convergence of the solution while avoiding large small-scale velocity variations.

## Results

After 10 iterations we achieved a significant variance reduction in P travel times from 0.480 s to 0.071 s (85%), and in S travel times from 0.767 s to 0.128 s (83%). The analysis of the resolving power of our dataset has been done empirically using the ray path coverage and synthetic reconstructions (spike tests). As expected, the results show good recovery beneath the island, where the ray path coverage is exceptional.

**Figure 4:**
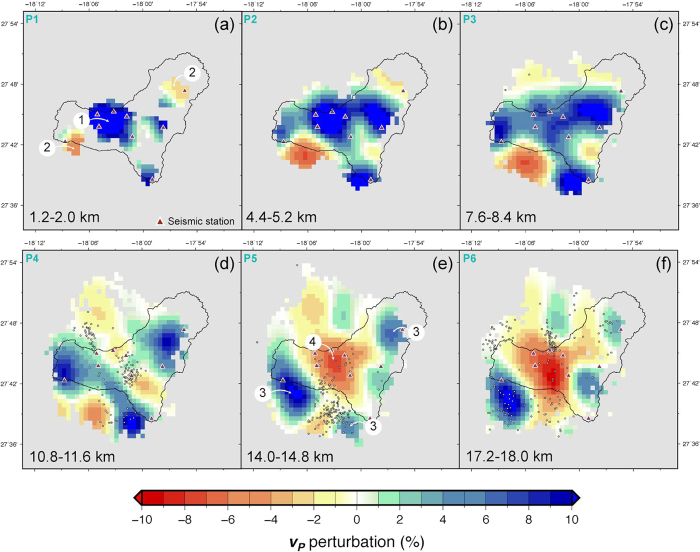
Horizontal slices through the final P-wave velocity model. *v*_*P*_ values are shown as percentage with respect to the velocity of the initial 1D model at each depth, according to the colour palette. Depth range of each map is indicated in the lower left corner. Seismic stations are shown as triangles, and earthquakes inside the depth limits of the horizontal slice are shown as small circles. Features discussed in the text are indicated by numbers inside white circles. Figure was created using GMT 5.3.1 software^55^ (http://gmt.soest.hawaii.edu/projects/gmt).

**Figure 5:**
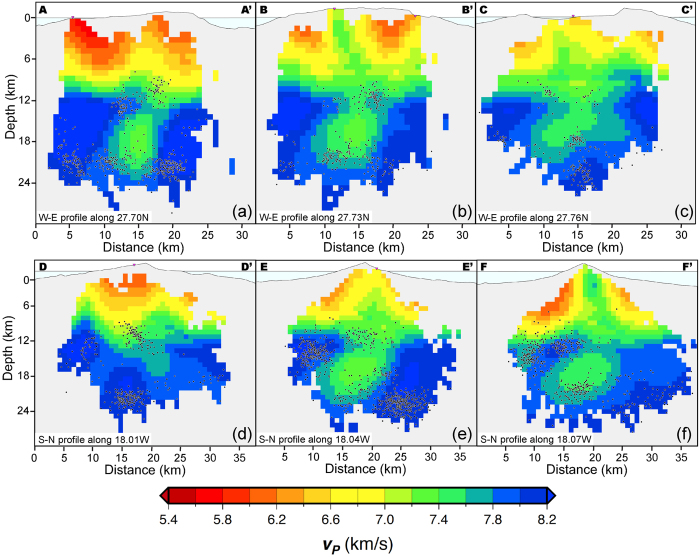
Vertical cross sections through the final P-wave velocity model (see Fig. 3 for location): (**a–c**) W-E oriented cross sections (W to the left and E to the right), and (**d–f**) S-N oriented cross sections (S to the left and E to the right). The latitude of the W-E cross sections, and the longitude of the S-N cross sections is indicated at the bottom of each panel. Earthquakes within 1 km of the cross section are shown as small circles. Figure was created using GMT 5.3.1 software^55^ (http://gmt.soest.hawaii.edu/projects/gmt).

The main results of this study are presented in Figs 4 and 5 in a set of horizontal and vertical sections. Figure 4 shows horizontal sections at different depths of the distribution of *v*_*P*_ perturbation (%) values, with respect to the 1D starting model. At shallow depths (1–2 km b.s.l.) it is remarkable the presence of a high *v*_*P*_ anomaly centred below the Tanganasoga volcano and extending across most of the El Golfo landslide valley (Fig. 4a, label 1). Other two less pronounced positive anomalies are located at the east and south of that main one (better seen in Fig. 4b), while two negative anomalies appear at the northeast and southwest of El Golfo anomaly (Fig. 4a, label 2). All these anomalies persist with depth, becoming more intense and better defined, except the negative anomaly located to the northeast, which dilutes progressively with depth. With increasing depth a new negative anomaly starts to become more visible in between three positive anomalies (13–18 km). At these depths also a new prominent positive anomaly appears at the western corner of the island. The El Golfo positive anomaly dilutes progressively when going deeper into the island structure, while the other three are better remarked. At a depth of 13–14 km the three positive anomalies, more or less located at three corners of the island, are maintained (Fig. 4e, label 3). At the same time, the central part is occupied by a large negative anomaly (Fig. 4e, label 4), which increases progressively in intensity with depth, while the positive anomalies located at the northeast and south of the island progressively disappear.

Figure 5 shows several N-S and E-W vertical sections of the distribution of *v*_*P*_ inside El Hierro. These sections reveal several important features of the anomalies shown before. The highest *v*_*P*_ values characterise the volume that forms the lower part of the volcanic edifice of El Hierro from 10–12 km depth b.s.l. This volume may show important discontinuities depending on the location of the vertical sections where it is visualised. At the limits of the island this volume is better defined and rather homogeneous, but towards the interior it becomes much more irregular with parts or apophysis entering into shallower levels, even reaching the surface at some points, at the time that a large anomalous body characterised by low *v*_*P*_ values occupies the centre of the island at depths between 12 and 22 km b.s.l.

## Discussion

As at the rest of the Canary Islands, the lithosphere at El Hierro volcanic edifice is composed of different main rock layers that from top to base include: the volcanic pile composed of an alternation of lava flows and pyroclastic deposits; a pre-volcanic sediment layer; the pre-volcanic basaltic oceanic crust; and the lithospheric mantle. In general terms, disparities between the Canarian volcanic edifices are marked by the different thickness of these main rock layers, the presence or not of lithospheric flexure, and the existence or not of underplating at the crust/mantle discontinuity^26^,^37^,^38^,^39^,^40^,^41^,^42^. In the case of El Hierro, bathymetric studies^41^,^43^, suggest that the volcanic pile is about 5,500 m thick, reaching 1,500 a.s.l. The pre-volcanic sediments would have a thickness of about 500 m, while the oceanic crust would extend down to 12–15 km depth and the lithospheric-asthenosphere boundary would be located at about 50–60 km^44^,^45^. No evidence of underplating at the crust/mantle discontinuity has been reported at El Hierro^9^,^40^,^44^, as well as no evidence of lithospheric flexure^39^.

Our study confirms this general lithospheric structure but allows going into more detail, thus distinguishing some important discontinues that reveal that the interior of El Hierro volcanic edifice is not stratigraphically homogeneous. The results obtained with this tomographic study are fully coincident with previous results from gravimetric^46^ and low-frequency microseismic sounding^47^. There is coincidence in the position and size of the gravimetric (high density/low density) and seismic (high velocity/low velocity) anomalous bodies, which reveal the location of the main magma intrusion and accumulation zones inside El Hierro, and the accumulation of lighter materials (e.g. pyroclastic deposits and porous lavas) during the construction of the island, as well as that of the main structural features. Our results are also similar to those of García-Yeguas *et al*.^48^ although they studied a smaller region using only data from the first unrest and the eruption, thus resulting in a smaller coverage in area and in depth.

**Figure 6:**
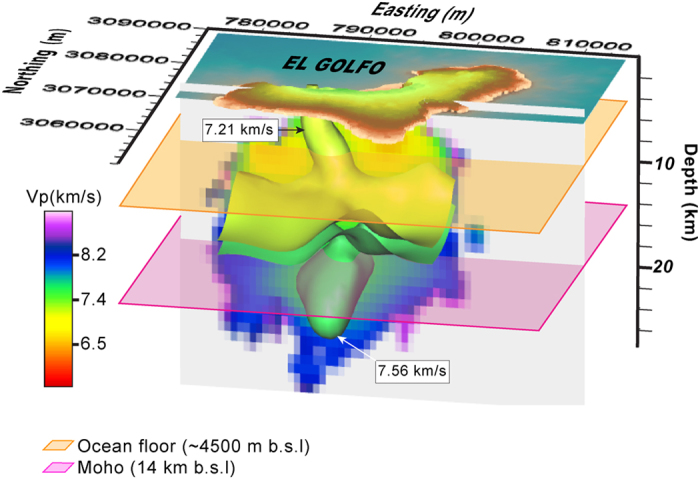
3D view of the P-wave model beneath El Hierro island showing the isosurfaces of 7.21 km/s and 7.56 km/s, which illustrate the shape of the upper-crustal Tanganasoga high-velocity anomaly, and the upper-mantle low velocity region corresponding to the magma reservoir, respectively. Figure created using Voxler 3.0 (Copyright *©* Golden Software, LLC) (www.goldensoftware.com/products/voxler).

As expected, *v*_*P*_ values increase with depth indicating a progressively higher density of the materials that form the El Hierro volcanic edifice, varying from 5.4 to 6 km/s at the volcanic pile, from 6 to 7 km/s at the oceanic crust, and from 7 to 8.2 km/s or higher at the lithospheric mantle. At the scale of observation, the pre-volcanic sedimentary layer is indistinguishable from the volcanic pile or from the crust. The distribution of *v*_*P*_ values is not laterally homogeneous inside El Hierro but shows significant discontinuities at different points where volumes of material with higher *v*_*P*_ replace zones of lower *v*_*P*_ and vice versa. We interpret these lateral discontinuities in the first case as representing main intrusion and accumulation zones of deep magmas, now already solidified, into the oceanic crust and the volcanic pile. These anomalous zones rise from the lithospheric mantle and some of them form apophysis that reach the surface at different points of the island, the most prominent being the one located below the Tanganasoga volcano at the interior of the El Golfo valley (Fig. 6). Other of these apophysis correspond to the former Tiñor volcano and to the southern end of the island. On the other hand, we observe the relative low velocity zone that occupies a significant volume located deeper (sub-crustal) in the centre of the island and that may correspond to a magma accumulation zone during the recent reactivation of El Hierro volcano. The geometry of this anomalous zones seems to be affected by the N-S main lithospheric structural feature identified by Carbó *et al*.^49^. It is also remarkable a less pronounced but still identifiable high velocity anomaly just below the crust/mantle discontinuity at the western side of the island, which could reflect underplating not having been identified till present (Fig. 6). From these results it is also worth mentioning that our data do not reveal any trace at depth of the postulated rift zones, so agreeing with recent field studies that suggest that these structures are rather superficial (<3 km) and that have only contributed to the lateral migration of magma when it reached close to the surface, not having interfered with deep magma transport^50^.

These discontinuities reflect structural and/or rheological contrasts (i.e.: stress variations) that have conditioned magma transport inside the island and, consequently, the evolution of the location of seismicity during the different unrest episodes that have occurred in El Hierro since 2011. In fact, during the pre-eruptive unrest episode at El Hierro (Fig. 1)^2^ magma migrated more than 20 km from north to south before reaching the point where it ascended to the surface, thus initiating the eruption on October 10, 2011. This lateral migration of magma took place at the crust/mantle discontinuity, a major rheological discontinuity that should favour accumulation and lateral migration of magma according to most theoretical models on magma emplacement^15^,^16^. From our tomographic results, this surface is rather heterogeneous showing a significant variation in depth, ranging from 11 to 15 km b.s.l., depending on the point of observation, which would explain the changes in the depth of the hypocentral location of seismicity observed during that unrest episode. The path followed by magma was probably controlled by one of the main lithospheric structural features of the island, oriented north-south, clearly defined by one of the main linear high-gravity gradients found in the Canaries^46^,^49^. However, at certain points the magma path was diverted turning around zones that now we have identified as high *v*_*P*_ (e.g.: dense) anomalous bodies connecting the lithospheric mantle with shallower levels inside the volcanic edifice. Magma only ascended to the surface when it found a suitable path for it, which corresponded to an east-west oriented strike slip fault that opened due to a combination of tectonic and magmatic stresses^51^,^52^. This is in agreement with the regional stress field assumed for El Hierro in which the maximum compressional stress is oriented approximately north-south and the minimum compressional stress is east-west^2^,^51^,^53^ and with the fact that the rift zones do not correspond to deep structures inside the volcanic edifice. An additional evidence of the shallow role of the rift structures is that it is now clear that magma ascended mostly aseismically for nearly 15 km - in about 30 h through a E-W regional fault. This fault has an orientation normal to that of the southern rift and was opened by an earthquake of magnitude 4.3 with a transcompressional focal mechanism^51^. When magma arrived at very shallow depths it was captured by a N-S fissure of the southern rift zone starting the eruption along that fissure^2^. This is in good agreement with one of the characteristics of monogenetic volcanism observed in other zones, which is that deep magma ascent is not controlled by the same fractures that will determine its final eruption at surface^54^.

Under this stress situation, however, why magma did not reach the surface earlier, when volumetrically significant eruptions had occurred during the Holocene along the same north-south lineation that was defined by the hypocentral location of the pre-eruptive seismicity? A simple explanation is that previous eruptions had sealed potential magma path fractures. In fact, one of the most important recent eruptive episodes corresponds to the Tanganasoga eruption^20^,^21^, which extruded a relatively large volume of dense ankaramitic (olivine-pyroxene rich) magmas, the remains of which could have blocked these previous eruption conduits. Actually, gravimetric studies^46^ and our tomographic results confirm that a dense body extending toward the base of the crust forms the core of the Tanganasoga edifice. This dense body would form now a stress barrier for the ascent of magmas along the same structural discontinuity. And the same situation would be repeated at the other anomalous bodies we have identified. An alternative or complementary explanation is that magma overpressure at that depth is not sufficient (probably because the total volume of magma was not large enough) to open by itself a full path to reach the surface if tectonic stresses do not play in its favour^52^.

On the other side, the north-south migration experienced by El Hierro magma before starting the eruption of 2011, seems that it was also controlled by a major structural feature that is clearly defined by one of the main linear high-gravity gradients found in the Canaries^46^,^49^, although the exact nature of such discontinuity has not been established yet. The post-eruption unrest episodes also show structural controls, as it is suggested by the coincidence of the location of seismicity and that of the main gravimetric and tomographic anomalies^46^.

## Conclusions

Tortuous dyke propagation events have been identified and studied in different volcanic environments, and have been interpreted by several authors to be directly driven and steered by principal stresses, as supported by theory. However, in the case studied here such tortuous pathways are also interpreted as caused by stress inhomogeneities controlled by inhomogeneous rock properties. The 3D seismic tomography conducted at the Hierro Island has revealed its internal structure and has permitted to identify the nature and location of the main rheological and structural discontinuities, which act as stress barriers affecting magma migration inside the crust. In fact, the unrest episode that preceded the eruption occurred in 2011–2012 was characterised by a tortuous dyke propagation^2^. Despite the regional stresses controlled the lateral movement of magma in all the recorded unrest episodes, the presence of specific stress barriers defined by rheological contrasts and structural discontinuities were able to divert magma movement at their vicinity, punctually changing the direction of propagation imposed by the principal stresses.

We presume that the situation at El Hierro may have also occurred in other volcanic systems where the presence of high density bodies and structural discontinuities inside the edifice may have obliged dykes choosing to ascend at the side of them. In this sense, the results obtained in our study recommend to check whether other well documented dyking events in which bending of the dyke pathway was also observed were constrained by blocked pathways rather than being only controlled by topographic and tectonic stresses (eg: Bardarbunga). Identifying such heterogeneities and anisotropies, through seismic tomography or other geophysical imaging techniques, may help determining the structures that control magma migration at a more local scale and to identify how magma has reached the surface. More generally, the application of such techniques in active volcanic systems may help to deduce the most probably pathways that magma will use to erupt at surface, so contributing to improve eruption forecasting. Obviously, the resolution of the imaging method will determine the dimensions of the structures we can identify. In our case, the resolution achieved has been sufficient to recognize some significant rock discontinuities inside El Hierro.

## Additional Information

**How to cite this article:** Martí, J. *et al*. Stress barriers controlling lateral migration of magma revealed by seismic tomography. *Sci. Rep.* **7**, 40757; doi: 10.1038/srep40757 (2017).

**Publisher's note:** Springer Nature remains neutral with regard to jurisdictional claims in published maps and institutional affiliations.

## Supplementary Material

Supplementary Information
