# Supplementary material for: Stress barriers controlling lateral migration of magma revealed by seismic tomography
Source: Sci Rep. 2017 Jan 13;7:40757. doi: 10.1038/srep40757 (PMC5233990; doi:10.1038/srep40757)
Supplement: Supplementary Information [file srep40757-s1.pdf]

Stress barriers controlling lateral migration of magma revealed by seismic tomography

Martí, J[1]., Villaseñor, A[1]., Geyer, A[1]., López, C[2], Tryggvason, A.[3]

1. Institute of Earth Sciences Jaume Almera, ICTJA-CSIC, Barcelona, Spain

2. Observatorio Geofísico Central, Instituto Geográfico Nacional (IGN), Madrid, Spain

3. Department of Earth Sciences, Geophysics, Uppsala University, Uppsala, Sweden.

Corresponding author: Joan Marti (joan.marti@ictja.csic.es)

Supplementary Material

1. Choice of the smoothing constraint

Here we describe how we determined the smoothing parameter *k* (see equation 7 in Benz et al. (1996) for its definition). This parameter controls the trade-off between the minimization of the data misfit and the model roughness. We have tried a wide range of smoothing parameters from very large (resulting in very smooth models) to very small (resulting in very rough models). Figure S1 shows the evolution of the RMS of the travel time residuals with each iteration for different values of *k*. Starting with the higher values, we can observe that, as expected, the RMS decreases continuously with each iteration, and also as we decrease the value of *k*. However, for values of *k* smaller than 50, the RMS value does not decrease significantly, and it even begins to oscillate instead of decreasing continuously. Therefore, we adopted the value of *k*=50 for our final model because it is the one that results in a smaller value of the RMS without degrading the quality of the model.

Figure S2 shows the effect the smoothing parameter on the velocity model. There we show three models obtained with values of *k*=100 (smooth, top panel), 50 (our preferred value, central panel), and 10 (rough, bottom panel). It is clear that the model obtained with a smoothing parameter of 10 is too rough, and unrealistic. On the other hand, models obtained with values of 100 and 50 show both the same features, although they seem to be better defined in the latter.

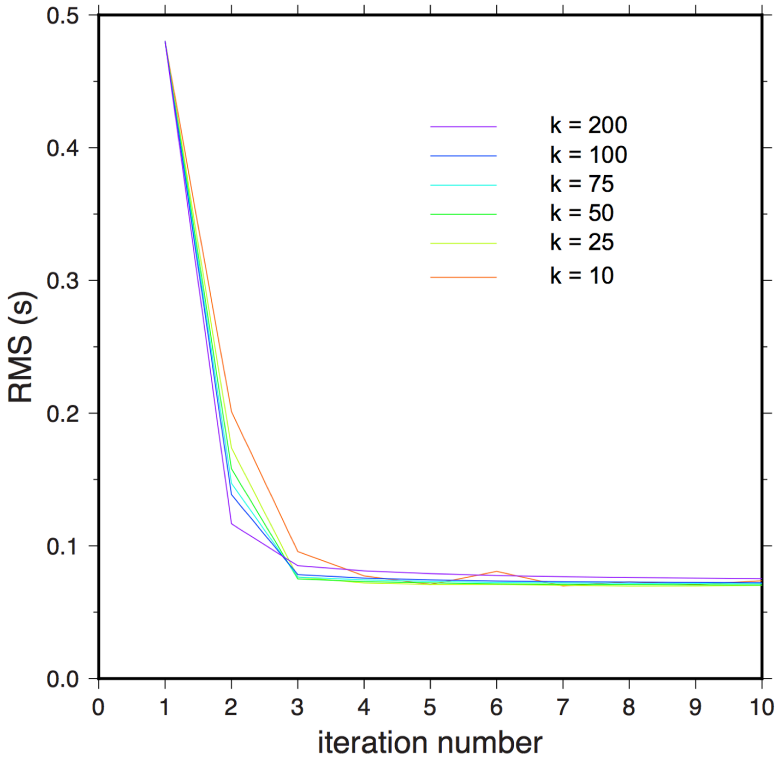

Figure S1. *Root mean square (RMS) of the travel time residuals as a function of iteration for different values of the smoothing parameter k. Values are indicated in the legend.*

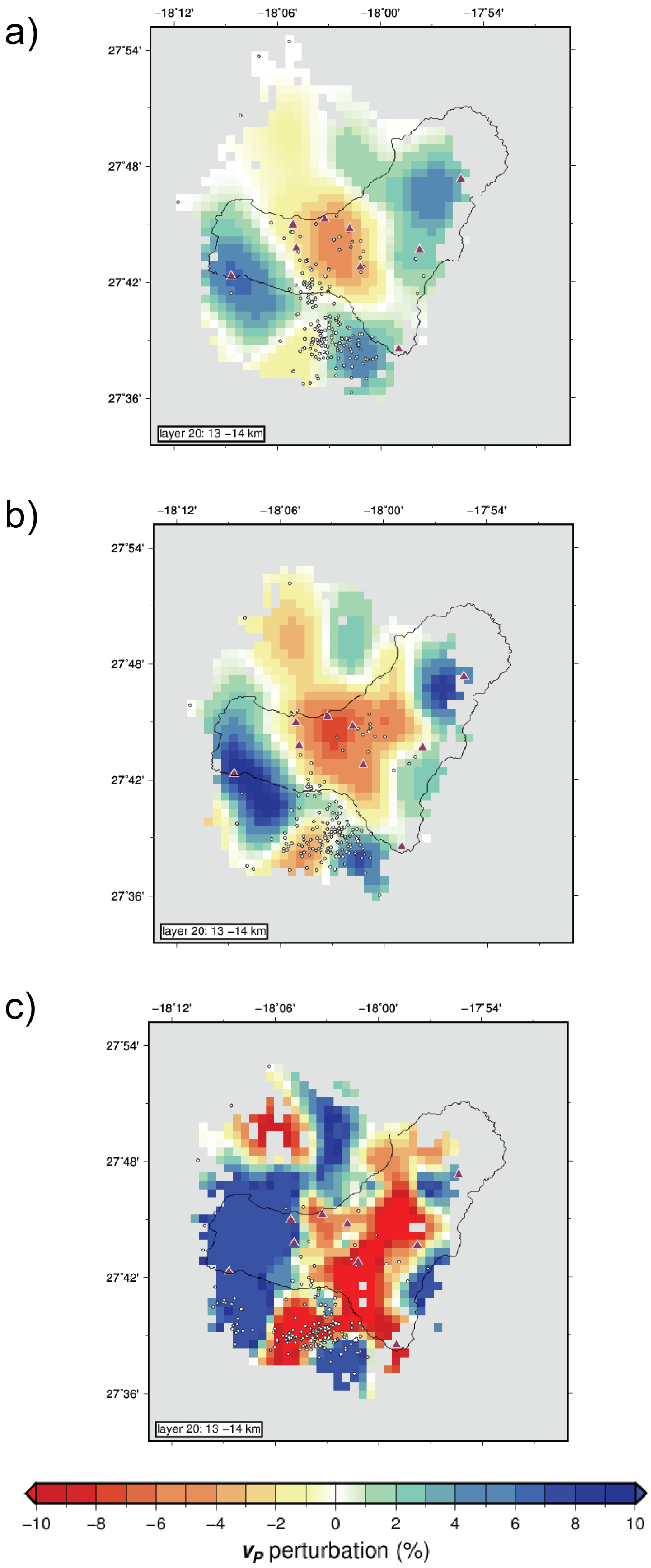

Figure S2. *Model results obtained using different values of the smoothing parameter for layer 20 of the model (13-14 km depth): a) smoothing parameter k=100 (overdamped). b) k=50 (value used for the model presented in the manuscript). c) k=10 (underdamped). Figure was created using GMT 5.3.1 software (Wessel et al 2013) (http://gmt.soest.hawaii.edu/projects/gmt)*

2. Assessment of the resolution of the P-wave model

The linearized tomographic inversion used in this study consists in solving a linear system of equations, that is, inverting a matrix. The LSQR solver used is an approximate, iterative method, and it does not determine the inverse matrix. Consequently, it does not allow for the computation of the resolution and covariance matrices. Therefore, the analysis of the resolution of the model is done empirically, using for example the ray-path coverage and synthetic reconstruction tests ("checkerboard" or "spike" tests).

For the synthetic reconstruction tests we use spike models, that are similar to traditional checkerboards, except that the alternating positive and negative anomalies are not adjacent, but separated by a padding with zero value of the anomaly. Rawlinson and Spakman (2016) have recently shown that spike tests provide better estimates of the range of scale-lengths that can be resolved than checkerboard tests.

Although widely used, checkerboard/spike tests only provide a qualitative estimate of the resolution of the model, and in some cases they can produce misleading results as shown by Leveque et al. (1993). Therefore, the results presented here cannot be directly related to formal resolution.

We have produced a synthetic spike model consisting of alternating positive and negative anomalies both in the horizontal and vertical directions. The spikes consist of 4 × 4 × 4 model cells with velocity anomalies of ±10% with respect to the initial model used in the inversion. Since the model cells are 0.8 × 0.8 × 0.8 km^3, the size of the spike anomalies is 3.2 × 3.2 × 3.2 km^3. Each spike is separated from the next by a buffer of two model cells (1.6 km) with 0% velocity anomaly.

We compute synthetic traveltimes through the spike model using the same forward method as in the tomography (Podvin and Lecomte, 1991) and for the same geometry of sources and receivers as in our arrival time dataset. We then invert this synthetic dataset using the same parameters (number of iterations, smoothing constraints) that were used to obtain our final P-wave model.

The results of the reconstruction are shown in Figures S3-S4. Each figure corresponds to a layer that intersects one of the spikes in depth. On the top panel is shown the synthetic spike model, and in the bottom panel the reconstruction obtained. In both panels cells that are not illuminated by any ray path are shown in gray. As expected in these tests, regions that are well covered by seismic stations and/or local earthquakes exhibit good reconstructions, while anomalies in poorly sampled regions (near greyed out areas) are not well recovered. Therefore, according with the spike tests presented her we can reliably interpret anomalies with size of $3.2 \times 3.2 \times 3.2$ km^3 and larger in the center of the model. The smoothing constraints imposed on the model prevent the full amplitude of the anomalies (10%) to be recovered, although close values are obtained in the best sampled regions. Similarly, since the high velocity spikes are better sampled than low velocity spikes, the anomalies of the former are better recovered than those of the latter.

In the discussion section have only interpreted features that occur in areas well reconstructed in the spike tests shown here, and that have at least a size of 4 model cells. Well sampled/reconstructed areas correspond basically to the emerged portion of El Hierro volcanic edifice.

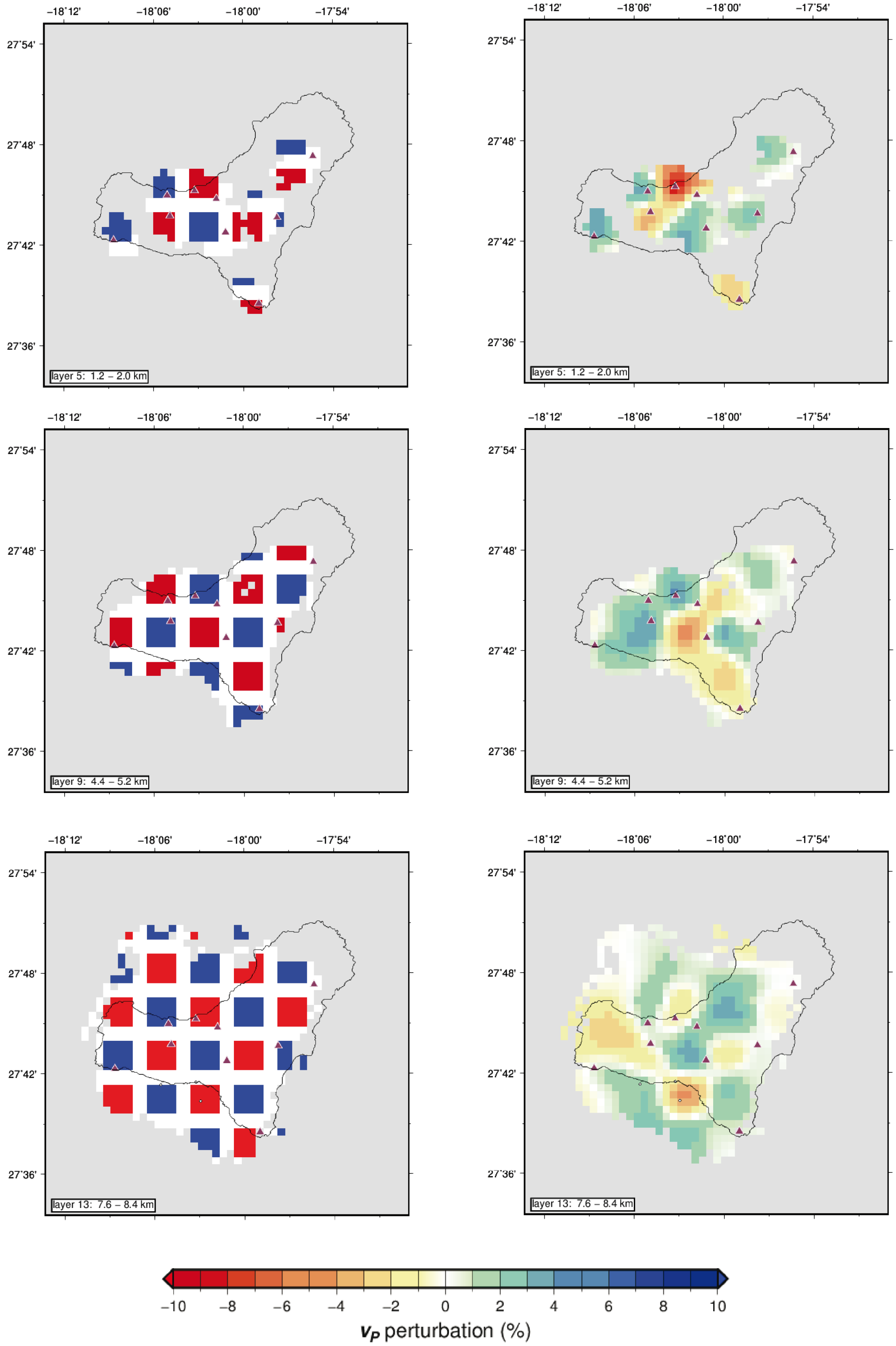

Figure S3*. Reconstruction tests for layers 5 (1.2-2.0 km depth, top), 9 (4.4-5.2 km depth, middle), and 13 (7.6-8.4 km depth, bottom) of the model. Left panels show the synthetic spike model for different layers, and the corresponding right panels show the reconstruction obtained with our traveltime dataset. Cells not sampled by rays are greyed out. Figure was created using GMT 5.3.1 software (Wessel et al 2013) (http://gmt.soest.hawaii.edu/projects/gmt)*

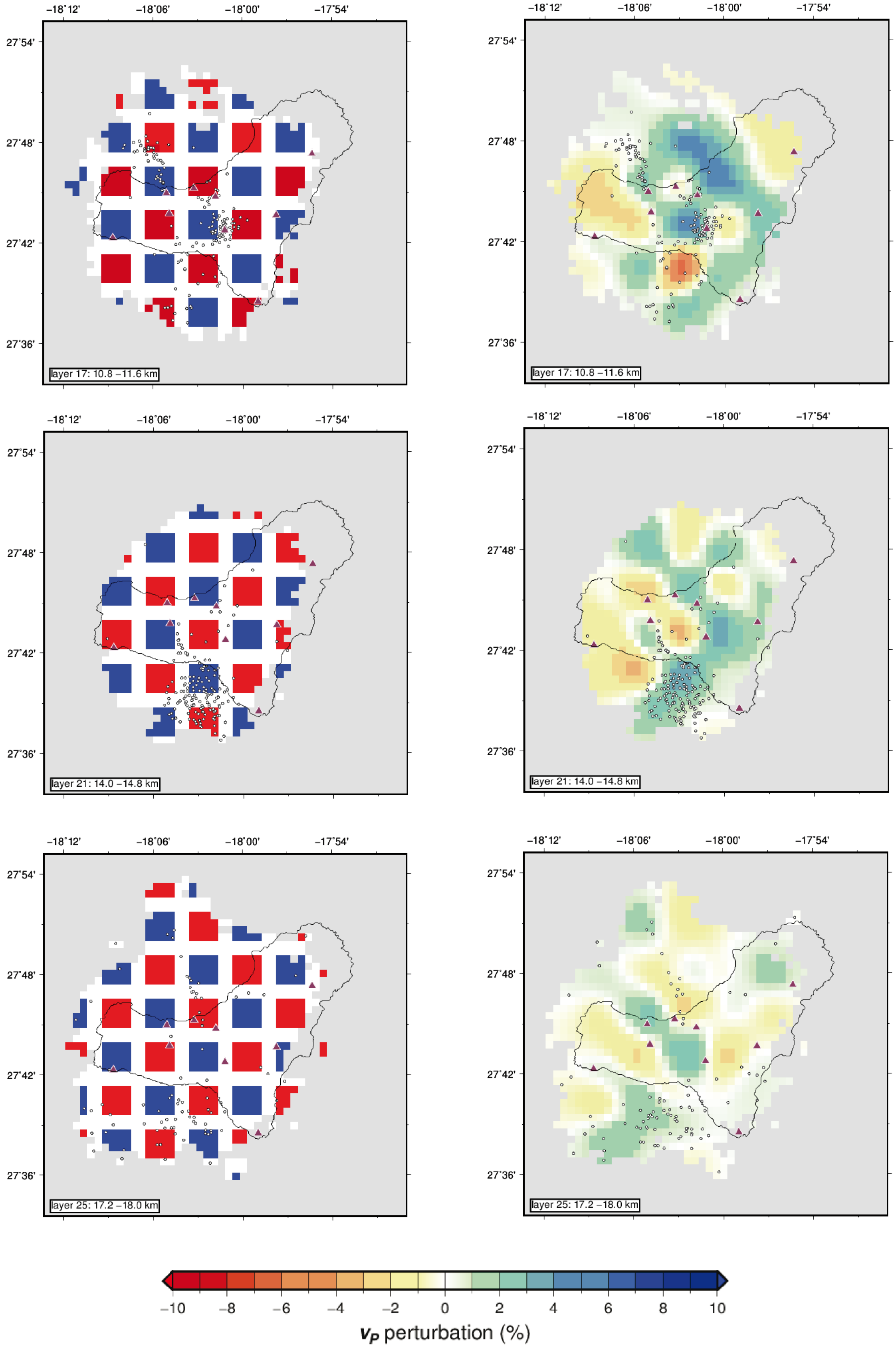

Figure S4. *Reconstruction tests for layers 17 (10.8-11.6 km depth, top), layer 21 (13.2-14.0 km depth, middle), and 25 (17.2-18.0 km depth, bottom) of the model. Left panels show the synthetic spike model for different layers, and the corresponding right panels show the reconstruction obtained with our traveltime dataset. Cells not sampled by rays are greyed out. Figure was created using GMT 5.3.1 software (Wessel et al 2013) (http://gmt.soest.hawaii.edu/projects/gmt)*

3. Comparison of the P- and S-wave velocity model

In our simultaneous inversion for velocity structure and earthquake relocation we obtain a P- and S-wave velocity model. In Figure S5 we show a comparison of both models for two horizontal layers. It can be observed that the major features are present in both models, including all those discussed in the manuscript. However, since the quality of the S-wave arrival time data is not as good as the P-wave data, we do not use the Vs model to define the different crustal and upper mantle bodies nor we discuss the distribution of Vp/Vs.

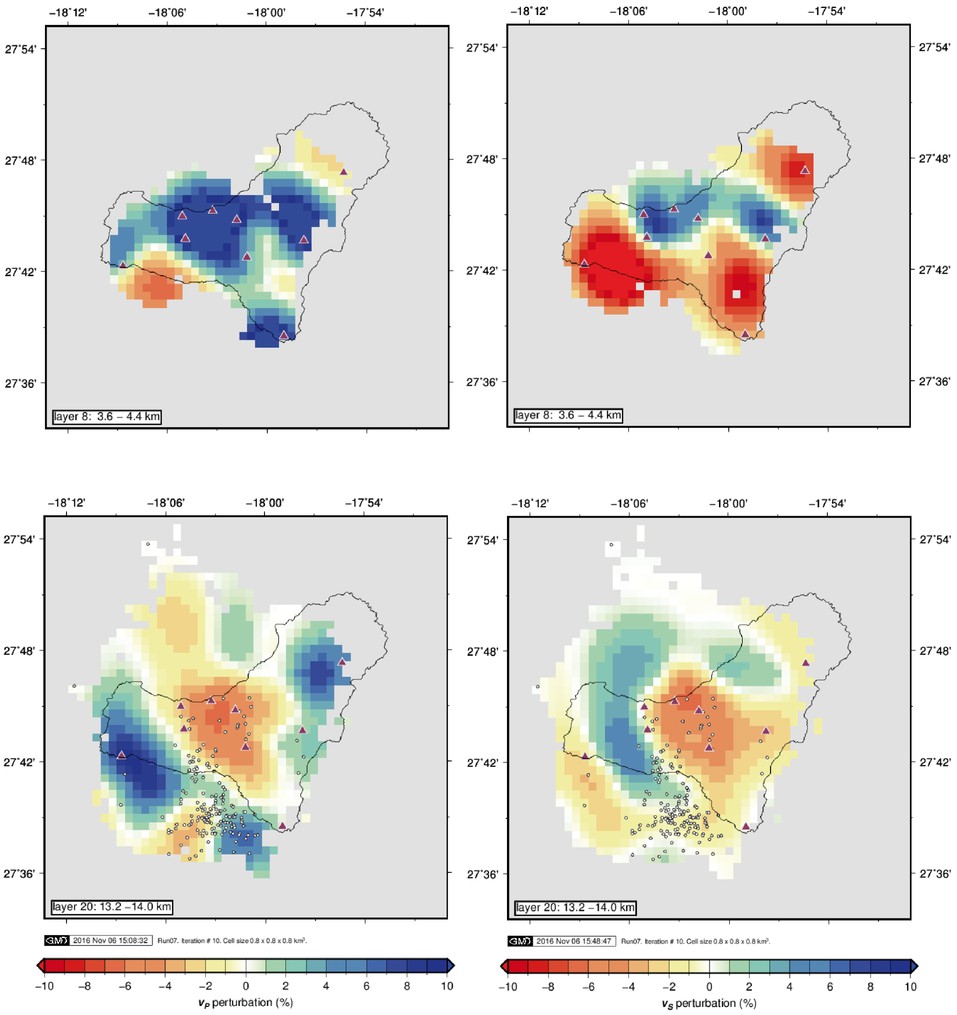

Figure S5*. Comparison of the P- and S-wave velocity models (left and right columns respectively) for two layers of the model. Top: layer 8 (3.6-4.4 km depth); bottom: layer 20 (13.2-14.0 km depth). The major features discussed in the manuscript are well imaged in both models. Figure was created using GMT 5.3.1 software (Wessel et al 2013) (http://gmt.soest.hawaii.edu/projects/gmt)*

REFERENCES

Benz, H.M., Chouet, B.A., Dawson, P., Lahr, J.C., Page, R.A., Hole, J.A., 1996. Three-dimensional P and S wave velocity structure of Redoubt Volcano, Alaska Journal of Geophysical Research 101, 8111-8128.

Leveque, J.J., Rivera, L., Wittlinger, G., 1993. On the use of checker-board test to assess the resolution of tomographic inversions. Geophysical Journal International 115, 313-318.

Podvin, P., Lecomte, I., 1991. Finite difference computation of traveltimes in very contrasted velocity models: a massively parallel approach and its associated tools. Geophysical Journal International 105, 271-284.

Rawlinson, N., Spakman, W., 2016. On the use of sensitivity tests in seismic tomography. Geophysical Journal International 205, 1221-1243

Wessel, P., W. H. F. Smith, R. Scharroo, J. Luis, and F. Wobbe, Generic Mapping Tools: Improved Version Released, EOS Trans. AGU, 94(45), p. 409-410 (2013). doi:10.1002/2013EO 450001ÿ
